# Frequency of red blood cell allo-immunization in transfused patients with sickle cell disease in Africa: a systematic review with meta-analysis

**DOI:** 10.4314/ahs.v24i3.46

**Published:** 2024-09

**Authors:** Theresa Ukamaka Nwagha, Angela Ogechukwu Ugwu, Martins Nweke

**Affiliations:** 1 Department of Haematology and Immunology, Faculty of Basic Clinical Sciences, College of Medicine, University of Nigeria, Ituku/Ozalla Campus Enugu Sttse, Nigeria; 2 Department of Haematology and Immunology, University of Nigeria Teaching Hospital, Ituku/Ozalla, Enugu State, Nigeria; 3 Department of Physiotherapy, Evangel University Akaeze- Okpoto, Ebonyi State, Nigeria; 4 Department of Physiotherapy, Faculty of Health Sciences, University of Pretoria, South Africa

**Keywords:** Systematic review, red blood cell, alloimmunization, sickle cell disease, Africa

## Abstract

**Background and Objectives:**

Blood transfusion is an effective and proven treatment for some severe complications of sickle cell disease. Recurrent transfusions have put patients with sickle cell disease at risk of developing antibodies against the various antigens they were exposed to. This study aims to investigate the frequency of red blood cell alloimmunization in patients with sickle disease in Africa.

**Materials and Methods:**

This is a systematic review of peer-reviewed and published literature. The review was conducted consistent with the Preferred Reporting Items for Systematic Reviews and Meta-Analyses checklist.

Data sources for the review include MEDLINE, PubMed, AJOL, CINAHL, Psych-Info and Academic Search Complete. Included in this review are articles that reported the frequency/prevalence of red blood cell alloimmunization in sickle cell disease patients in Africa. Eligible studies were subjected to independent full-text screening and data extraction. Risk of bias assessment was conducted with the aid of the mixed method appraisal tool. We employed a random-effects model of meta-analysis to estimate the pooled prevalence. We computed Cochrane's Q statistics and I2 and prediction interval to quantify heterogeneity in effect size.

**Results:**

The prevalence estimates range from 2.6% to 29%. Pooled prevalence was estimated to be 12.1% (95% CI 8.1 to 17.7), with significant heterogeneity (I2= 91.83; PI = 2 to 54%).

**Conclusion:**

The frequency of red cell alloantibody varies considerably in Africa.

## Highlights

- The prevalence of alloimmunization among sickle cell anaemia patients varies considerably in Africa. The prevalence estimates range from 2.6% to 29%
- There is a dire need for carrying out an extended antigen typing for the donors and for the sickle cell patients themselves. This will serve to provide available compatible blood units for blood transfusion

## Introduction

Chronic anemia and painful crises are common symptoms in Sickle Cell Disease (SCD), which are genetic abnormalities defined by the presence of a mutant type of hemoglobin S (HbS)^1^. Nearly a quarter of a million people are diagnosed with SCD every year^2^. Africa, India, and the Middle East are among the regions with the highest prevalence of SCD^3^. Three-quarters of all sickle cell cases are found in Africa^4^, with Nigeria representing the highest prevalence of the world's SCD occurrence^5^. Through the ensuing adherent erythrocytes subject to lysis, these genetic mutations contribute to the severity of SCD and clinical symptoms including vaso-occlusive crises, acute chest syndrome, priapism, pulmonary hypertension and leg ulcers seen in patients with SCD^6^,^7^. Treatment modalities for SCD are either disease-modifying, preventative, or symptom-focused. Disease-modifying drug therapy such as hydroxyurea is now available for many African patients. However, HU reduces pain crises and severe complications but does not eliminate the need for transfusion^8^-^10^.

Currently, allogeneic bone marrow transplantation (BMT) and gene therapy and gene editing using CRISPR Cas-9 have become treatment options that can cure sickle cell disease^11^-^13^ although, for the large majority of SCD patients in Africa these are not readily available.

Red cell transfusion is a standard practice in the supportive care of individuals with SCD^14^. Transfusions are critical in the prevention and treatment of chronic SCD complications, such as stroke, chronic kidney disease and acute chest syndrome^14^. Blood transfusion is associated with several complications: risk of transfusion transmissible infections, iron overload, haemolytic transfusion reactions and alloimmunization^15^-^17^. These complications diminish the benefits of blood transfusion.

Alloimmunization is a common side effect of recurrent blood transfusions^18^. Compared to the 2-5% rate observed in general transfusion recipients^19^, red blood cell alloimmunization occurred in 20% to 50% of transfused patients with SCD who were matched for ABO and D antigens only^20^. Various studies have established the prevalence of red blood cell alloimmunization in African patients with blood cell disorders^21^-^25^. Prevalence rate ranges from 1.1 % in Malawi to 22 % in South Africa^21^, ^22^.

Alloimmunization develops when the recipient is exposed to donor red cell antigens which are absent in the recipient^26^. The antibodies produced are then directed against the recipient's red cells resulting in transfusion reactions that are potentially serious. Several factors have been found to contribute to the high rate of alloimmunization among patients with SCD^27^. These include discordance of blood group antigen expression on donor and patient RBCs, degree of antigen matching and increasing number of red cell transfusions^28^. Following exposure to transfused red cells, not all patients develop alloantibodies. This observation was noticed not only in patients with SD but also in all transfused recipients. Red blood cell alloimmunization-related problems include difficulty finding compatible blood units for these patients, delays in transfusions due to the presence of alloantibodies, and delayed hemolytic transfusion reactions^29^-^31^. Common RBC antigens implicated in alloimmunization in SCD include C and E in the Rhesus (RH) blood group, K in the Kell (KEL), Fya in the Duffy (FY), Jkb in the Kidd (JK), and S in the MNS blood groups^20^. In order to prevent alloimmunization, extended antigen matching beyond ABO and RhD are employed especially in chronically transfused patients although its use is severely limited by cost^31^. Alloimmunization management is expensive especially in Africa where only a few patients can afford treatment^31^. Therefore, in order to attain Universal Health Coverage and the African Union Agenda 2063^32^, it is vital to aggregate the frequency and antibody specificities in Africa, providing stakeholders with the drive to undertake large-scale serologic matching for Africans with sickle cell disease. A systematic review and meta-analysis by Boateng et al in 2019 reported a prevalence estimate of 7.4%^33^. However, it was limited to sub-Saharan Africa. To ensure inclusiveness, our review aimed at synthesizing the frequency of red blood cell alloimmunization in patients with sickle cell disease in Africa. This review covered the period up to October 2023.

## Materials and methods

This is a systematic review of observational studies of a cross-sectional or longitudinal design. We structured the protocol following the Preferred Reporting Items for Systematic Reviews and Meta-Analyses (PRISMA) guideline^34^.

### Eligibility criteria

#### Study characteristics

This review included peer-reviewed literature irrespective of sample size, and test statistics. Participants in the included studies were individuals diagnosed with SCD. Studies were included without regard to whether a control group was used to study the subject in question, provided they investigated the frequency of red blood cells alloimmunization in sickle cell disease patients.

Studies included in this systematic review were non-interventional by design.

### Inclusion criteria

Peer-reviewed articles reporting frequency of red blood cells alloimmunization in sickle cell disease patients in Africa.

1. Published peer review literature in English language and French
2. Studies were included irrespective of country, patient age, gender, and date of publication

### Exclusion criteria

1. Abstract-only papers as preceding papers, conference, editorial, and an author response
2. Studies with duplicate or overlapping data

### Information sources and search strategy

The search was undertaken of four databases namely; PubMed, MEDLINE, CINAHL, Psych-Info, African Journal online (AJOL) and Academic Search Complete using medical subject headings (MeSH), and keywords identified in the title, abstract, and/or text of the articles. The search strategy was piloted in PubMed. Included in the pilot search were MeSH terms identified through the Cochrane MeSH term. After several combinations of these terms, the most sensitive strategy was chosen and reported. Judgment of sensitivity was based on counting the number of potential relevant articles in the first few pages of the preliminary search result. The strategy was adapted to the syntax and subject headings of the remaining databases (MEDLINE, CINAHL, and Academic Search Complete). The search terms used in the search query included: ((Sickle Cell Anemia OR Hemoglobin S Disease, Hemoglobin S OR Sickle Cell Anemia OR Sickle Cell Disorders, Sickle OR Sickling Disorder Due to Hemoglobin S OR HbS Disease OR Sickle Cell Disease, Sickle OR Sickle Cell DiseasesMeSH Terms) AND (alloantibodies or alloantibody or alloimmunization or red blood cell alloimmunization) [All Fields AND (Africa or sub-Saharan Africa)[All fields. The initial search was conducted through 10th January 2022 and later updated in October 2023. The reference lists of selected articles and reviews were searched for possible identification of relevant studies.

### Study records and data management

Literature search results were exported directly from databases into EndNote 8 where de-duplication and initial screening were carried out. To aid the screening process, we used an already piloted and refined screening template that includes eligibility questions.

### Selection process

**Figure 1:**
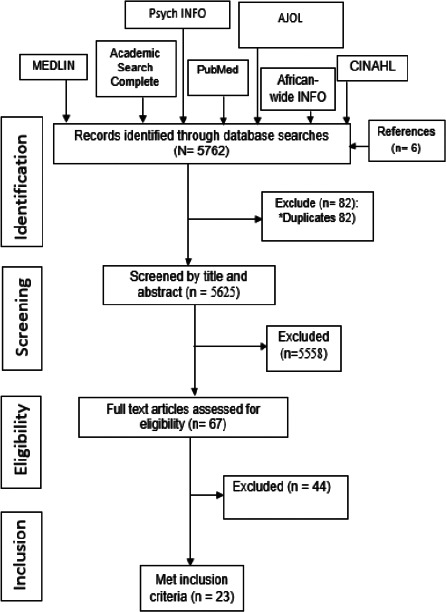
PRISMA Flow Diagram of the systematic review “Frequency of red cell alloimmunization and antibody specificities among patients with sickle cell disease in Africa (1994-2022)

Initial screening of the title and abstract to identify those that met the inclusion criteria was undertaken by MN. This was followed by independent screening by two research assistants MU and EN. The full texts of the included articles were downloaded by one of the research assistants. Independent screening of full texts and data extraction were conducted by MU and EN and conflicts were resolved by consensus discussions. Where appropriate, emails were sent to authors of selected studies to clarify issues pertaining to selection criteria. Details of the flow of studies throughout the selection process, along with the reasons for exclusion, were presented using a PRISMA diagram (Figure 1).

### Data collection process

#### Quality appraisal and risk of bias assessment

We used the mixed method appraisal tool (MMAT) Version 2011 to critically evaluate the studies we chose^35^. The MMAT looks at the study's purpose, adequacy, and methodology, as well as the study's design, participant recruitment, data collection, data analysis, presentation of findings, and discussions and conclusions. Quality appraisal was independently executed by two the authors (TUN) and (AOU) with an excellent inter-rater agreement. The disagreements were resolved by a third author (MN).

#### Data items

Primary data sought include the frequency of red blood cell alloimmunization in sickle cell disease patients in Africa. Secondary data include age, gender and rate of transfusion. Other information gathered from each article includes the authors' name, study title, study population, study sample size, sampling methods, method of assessment, prevalence rate, the antibody specificities, country, African region and summary of findings.

#### Data synthesis and assessment of heterogeneity

Using a random-effect model, the pooled prevalence was estimated in the manner described by Wang and Liu^36^. The heterogeneity measure, statistics, I2 was calculated following Higgins et al.^37^. I2 values were interpreted per the Cochrane Handbook for Systematic Reviews of Interventions as follows: 0–40% may indicate low heterogeneity, 30–60% may indicate moderate heterogeneity, 50–90% may indicate substantial heterogeneity, and 75–100% may indicate considerable heterogeneity^37^. This is shown in Table 2. Given that I2 is not an absolute measure of heterogeneity, we computed prediction intervals (PI) (Figure 5). Where appropriate, we employed Hozo et al.^38^ to compute mean from range, median and sample size.

#### Data analysis

The frequency of red blood cell alloimmunization in patients with sickle cell disease was estimated in percentages. We used meta-regression to examine factors which contributed to study heterogeneity. We employed analysis of covariance (ANOVA) to compare the prevalence of alloimmunization across year of publication. Firstly, we checked for possible correlation relationship percentage female gender, age, region sample size with frequency of alloimmunization. Since we found no statistical significant relationship between the sample factors and frequency of alloimunization, we ran a simple ANOVA and reported the mean plot. Comprehensive Meta-analysis software was used, with a set at 0.05.

#### Publication bias and sensitivity analysis

To examine metabias, we created a funnel plot and ran the Egger's regression test^39^ and the analysis of the frequency of alloimmunization was done.

## Results

A total of 5762 records were identified from databases, and reference lists. Following de-duplication, we eliminated 82 records, leaving 5625 articles for the title and abstract screening. Ultimately, 23 studies met the eligibility criteria (Figure 1). The 23 studies were also included in the meta-analysis^22^, ^23^, ^25^, ^33^, ^40^-^58^.

### Sociodemographic Characteristics and Study Quality

Eighteen studies were from SSA, with 6 (33.3%) of the articles being conducted in Nigeria. Five of the studies were conducted in 3 non-SSA African countries (Sudan, Tunisia and Egypt). A total sample of 2961 SCD patients with varying transfusion rates were involved. All 2961 patients were involved in the prevalence estimates for alloimmunization. Antibody specificities were reported for 21 studies with a total of 2748 patients; three studies namely Elkobani et al.^49^ and Hmida, 1994^50^ with sample sizes of 31 and 183 respectively did not report antibody specificities at all or did not report specificities for SCD particularly. A total of 262 patients experienced alloimmunization. We summarised sex distribution based on 20 studies and age based on the 23 studies. The mean age of the study participants was 16.3 ± 9.0 years. The male to female ratio was 1.2 (60%: 50%.)

Although all the participants included in the meta-analysis had been transfused at least once, there was insufficient detail to determine the number of transfusion per patient (Table 1). Regarding study quality, 18 (78.3%) of the studies were at low risk of bias (Supplementary Appendix S1).

**Table 1:** Study and transfusion characteristics of alloantibodies in sickle cell disease

	Authors[ref	Country (region)	No of patients in the entire study/no of transfused patients	Age (years) Mean (SD; range)	RBCc transfused Mean (SD; range)	Male to Female ratio	No. of patients with alloantibodies (%; 95% CI)	Number of sickle cell patients	Antibody test method	No. of alloantibodies: antibody specificities in sickle cell disease
1	Abbas et al^40^	Sudan in North Africa	100/100	4.13	6.73 (SD, 4.46; NR).	1.33	4 (4.0; NR)	100	Saline method	4: 2 K, 1 C 1 E
2	Adewoyin et al.^41^	Nigeria in West Africa	80/80	Mean 27.92 (8.82; <20-<20)	NR	1.05	6 (7.5; NR)	80	Tube agglutination with bovin albumin enhancement	8: 3 E, 2 C, 1 D, 1 Lea and 1 UI
3	Adewoyin et al. 2016^42^	Nigeria in West Africa	55/41	Mean 23*(128; 2-51)	Mean 4.5; (7.6; 1-55)	1.39*	4 (9.8; 3.2-24	41	TT (E-NR)	6: 2 C, 2 E, 1 k, 1 Lea
4	Akpan et al.^43^	Nigeria West Africa	80/80	Mean 29.0 (5; NR)	NR	1.42	9 (11.3; NR)	80	Panel of cells+Albumin(.ICT)	9: 2 E, 2 C, 1 D, 1 e, 1 K, 1 Kpa, 1 Lu
5	Aly et al.^23^	Egypt in North Africa	42/42	Mean 7.4 (3.32; NR)	3.5 (NR; 4-11)	1.10	9 (21.4; NR)	42	ID.Card micro-typing system	9: 3 K. 2 E, 2 C 1 Le' and 1 Jk'
6	Batina Agasa et al.^44^	Congo in Central Africa	144/127	Mean 15.5 (11.1; NR)	Mean 5.3 (6.6; 1-40)	NR	13 (10.2; 5.6-17)	127	CGA-LISS	17: 3 D, 3 C, 2 E, 9 UI
7	Boateng et al.^33^	Ghana in West Africa	154/154	Mean 10.65 (7.3; NR)	Median 2-4 (NR; 1-10)	1.30	10 (6.5; 3.3-11.9)	154	CGA-LISS	13: 3 D, 3 M, 2 E, 2 C, 1 e, 1 Cw, 1 UI
8	Boma Muteb^46^	Congo in Central Africa	39/39	Mean 8.6 (6.4; NR)	82% received 2 U (NR; NR)	0.77	1 (2.6; 0.1-15.1)	39	NR	1: 1 K
9	Diarra^47^	Mali in West Africa	133/90	Mean 21* (NR; 1-62	NR	0.73	4 (4.4; 1.4-12)	90	CGA (E-NR)	4: 2 C, 1 D, 1 c
10	Eldour et al.^48^	Sudan in North Africa	210/210	Median 2-5 (NR; 2-20)	≥2 U (NR; NR)	1.44	9 (4.3; 2.1-8.3)	210	Saline method	10: 5 K, 2 E, 1 c, 1 JK, 1 Leb
11	Elkobani et al.^49^	Sudan in North Africa	97/97	Mean 24.74 (18.2;NR)	Median 2-4 (2-4 to >20)	1.6	9 (29.0; NR)	31	Gel card system	NR
12	Hmida et al. 50	Tunisia in North Africa	309/182	Mean 12.3 (<5 to >10)	NR	NR	13 (7.14	182	-	NR
15	Kambale-Kombi et al.^51^	Congo Central Africa	261/261	Median 16.6 (NR: 9.3-23.)	Mean 6.5 (NR; 3-93)	1.23	43 (16.5; NR)	261	Gel cards system	72: 6 D, 7 C, 7 E, 1 c, 3 Cw, 3 K, 3 Fya, 2 Fyb, 1 Fy3, 2 Jkb, 17 M, 8 S, 3 U, 6 Lea, 2 Leb, 1 UI
13	Kangiwa et al^52^	Nigeria in West Africa	120/80	Median *21 (NR; 1-50	Mean 3 (NR; 2-25)	0.78	15 (18.8; 11-29)	80	CGA-LISS and enzymes	13: 2 E, 2 c, 2 e, 1 D, 1 Fya, 1 Fyb, 1 k, 1 Kpb, 1 Jsb, 1 Lua
14	Kuliya-Gwarzo^53^	Nigeria in West Africa	135/68	Mean 13.9 (6.5; NR)	Median 1-5 (NR; 1-10	1.52	6 (8.8; 3.6-18.9	68	TT-Alb and CGA	11: 2 D, 2 E, 1 Kpb, 1 Jsb, 1 Wra 1 Mg, 1 Vw, 1 Dia, 1 Goa
16	Mangare et al^24^	Kenya in East Africa	137/137	Mean 8 (NR; NR)	Mean 2.4 (NR; 1-8)	1.02	4 (2.9; 0.9-7.8)	137	CGA-NaCl/LISS	4: 1 Cw, 1 S, 1 M, 1 Cob
17	Meda et al^54^	Tanzania East Africa	365/365	Mean 16.49 (8.38; NR)	Mean 3.2; (NR; 1-40	0.99	15 (4.1; 2.4-6.8)	365	TT-NISS	63: 12 K, 9 Lea, 5 Cob, 4 Fyb, 4 Kpa, 3 D, 3 E, 3 Cw, 3 Lua, 2 C, 2 Leb, 2 S, 2 s, 2 Jkb, 2 P, 1 M, 1 N, 1 Jka, 1 PAN, 1 UI
18	Natukunda et al^25^	Uganda in East Africa	428/428	Mean 12 (10.5; NR)	Median 3 (NR; 2-100	1.04	26 (6.1; 4.1-8.9)	428	CGA-LISS	32: 10 E, 7 D, 4 S, 2 C, 2 Jka, 2 PAN, 1 K, 1 Fya, 1 Lea, 1 Cw, 1 M
19	Sekongo et al^55^	Côte D'Ivoire in West Africa	42/42	Mean 24.5 (16.0; NR)	Mean 9 (NR; 1-22)	1.0	12 (28.6; 16-45)	42	CGA-LISS	14: 6 E, 4 C, 1 D, 1 e, 1 S, 1 UI
20	Siransy et al. 2018^56^	Côte D'Ivoire in West Africa	31/27	Mean 21.6 (11.9; NR)	Mean 5.7t (NR; 1-N10)	0.82	5 (18.5; 7.0-39)	27	CGA (E-NR)	3: 1 C, 1 E, 1 Lea (specificity was determined in 2 patients)
21	Tebuka et al^57^	Tanzania In East Africa	200/200	Mean 5.6 (4.36; NR)	Median 3-4units (2U to ≥	1.17	17 (8.5; NR)	200	ICT+panel of cells	23: 6 C, 3 c, 2 E 1 e, 6 K, 2 M, 1 Fya, 1 JKa, 1 Le
22	Thompson et al^22^	South Africa in Southern Africa	182/91	Mean 20.9 (12.5;4-55)	Mean 2.77 (NR; 1-17)	NR	20 (22.0; NR)	91	AutoVue/Bio Vue	21: 8 E, I E, 3 C, I c, 3 K, 2 S, 1 s, 1 Fyb & 1 UI
23	Ugwu et al^58^	Nigeria in West Africa	86/86	Mean 26 (7.4; NR)	≥2 (NR; 2-NR)	1.10	8 (9.3; 4.4-18)	86	CGA-LISS	11: 3 E, 2 C, 1 D, 1 e, 1 K, 1 Kpa, 1 Jsb, 1 Lub

* Including the non-transfused SCD patients because data for transfused patients were not reported separately.

Column gel agglutination low ionic strength saline (CGA LISS)

TT: NISS: tube test Normal ionic strength saline

ICT: indirect Coombs test

TT: Alb tube test albumin

NR - no report

E - NR: antigen E no report

UI - Unidentified

**Appendix S1:** Assessment of risk of bias using the mixed method appraisal tool

Author's ID	Sampling strategy relevant?	Is the sample representative of the target population?	Are the measurements appropriate?	Is the risk of non-response bias low?	Statistical analysis appropriate?	Remark
Abbas et al 2013	Yes	yes	yes	yes	yes	Low risk
Adewoyin et al. 2021	No	Yes	Yes	Yes	Yes	Low risk
Adewoyin et al. 2015	No	Yes	Yes	Yes	Yes	Low risk
Akpan et al. 2022	No	Yes	Yes	Yes	Yes	Low risk
Aly et al. 2012	No	Yes	Yes	Yes	Yes	Low risk
Batina-Agasa et al. 2010	No	yes	yes	yes	yes	Low risk
Boateng et al. 2016	No	Yes	Yes	Yes	Yes	Low risk
Muteb et al. 2017	No	No	Yes	Yes	Yes	Moderate risk
Diarra, 2013	Yes	No	yes	yes	yes	Low risk
Eldour et al. 2015	Yes	Yes	Yes	Yes	Yes	Low risk
Elkobani et al 2020	No	No	Yes	Yes	Yes	Moderate risk
Hmida et al. 1994	No	IS	Yes	Yes	Yes	Moderate risk
Kangiwa et al 2015	No	Yes	Yes	Yes	Yes	Low risk
Kuliya-Gwarzo	IS	IS	IS	IS	IS	IS
Kambale-Kombi et al. 2023	No	Yes	Yes	Yes	Yes	Low risk
Mangare et al 2015	No	Yes	Yes	Yes	Yes	Low risk
Meda et al 2014	No	Yes	Yes	Yes	Yes	Low risk
Natukunda et al 2010	No	No	Yes	Yes	Yes	Moderate risk
Sekongo et al 2017	Yes	Yes	Yes	Yes	Yes	Low risk
Siransy et al. 2017	No	Yes	Yes	Yes	Yes	Low risk
Tebuka et al 2020	No	Yes	Yes	Yes	Yes	Low risk
Thompson et al 2019	Yes	Yes	Yes	Yes	Yes	Low risk
Ugwu et al 2015	No	Yes	Yes	Yes	Yes	Low risk

IS: insufficient information

### Prevalence and antibody specificities of alloimmunization in sickle cell disease in Africa

**Figure 2:**
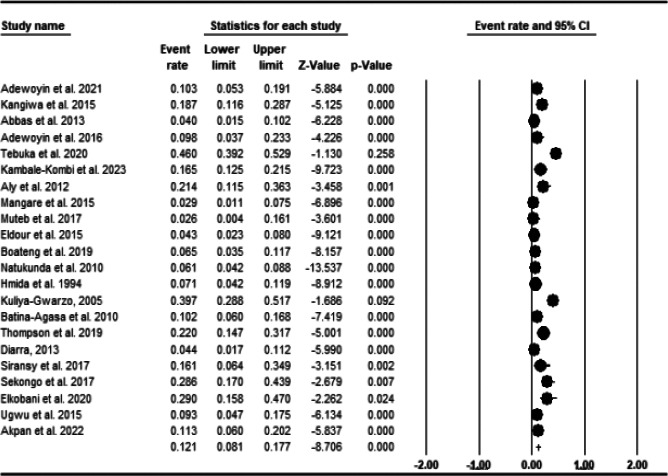
Forest Plot displaying the frequency of red cell alloimmunnizationhe amongst patients with Sickle cell disease

**Figure 3:**
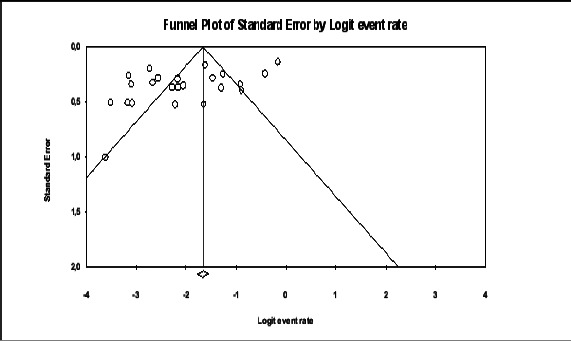
Funnel plot depicting publication bias

A total of 2961 individuals participated in the studies included in this review, with an average of 4.9 units, out of which 262 had alloimmunization. The prevalence estimates range from 2.6%^46^ to 29%^49^. Pooled prevalence was 12.1% 95% CI 8.1 to 17.7; PI = 2 to 54%); with a significant heterogeneity (I2=91.83) and publication bias (Egger's t = 2.685, p= 0.0139) (Figures 2 and 3). Between year 2005 and 2023, the frequency of alloimmunization varied considerably (Figure 3).

Analysis of variance was ran to account for the putative cause of heterogeneity. Result shows there was a near-double increase in the frequency of alloimmunization increased moving from before year 2010 (8.0%) to 2015-2020 (15.0%). Studies with small size sample size (<50) reported higher frequency of alloimmunization compared to those with sample size >50. Epidemiologically, the frequency of alloimmunization in Eastern Africa (8.0%) differed from those of Northern (13.2) and West Africa (11.8%). Epidemiologically, the frequency of alloimmunization was lowest with the Column gel agglutination low ionic strength saline (CGA LISS) test method (4.2%) and highest in highest with indirect Coombs test (ICT) method (28.6%). We observed a moderate positive correlation between age and frequency of alloimmunization. There was no correlation between gender and frequency of alloimmunization (Table 2). The extent of which the variance in age, sample size, region and antibody method and year of publication explained the total variance in true effect (1.24) could not be ascertain as multivariate meta-regression could not be ran due to collinearity associated with some of the characteristics (Table 3).

**Table 2:** Meta-regression showing the degree of heterogeneity explained by the difference in study characteristics

Characteristics	Odds ratio	P-value	Q-statistic	R^2^ (coefficient of determination)	% total variance (1.157) explained by the model	Number of studies included in the analysis
Female-male ratio						
	0.797	0.478	0.00	0.00	0.00	18
Age						
	0.0077	0.818	268.40	0.00	0.00	18
Sample size						
Sample size ≥50 (ref)	1.152	0.124	0.12	0.00	0.00	18
Sample size <50						
Region						18
Central Africa (ref)	ncc	ncc	ncc	ncc	ncc	
East Africa						
North Africa						
Southern Africa						
West Africa						
**Antibody test method**						
CGA (ref)	ncc	ncc	ncc	ncc	ncc	18
Saline						
Tube agglutination						
ICT						
Gel Card system						
ID Card micro-typing						
Aut0Vue Bio						

*significant at α < 0.01; ref: reference category; ncm: not computed due to colinearity

**Table 3:** ANOVA showing the influence of study characteristics on frequency of alloimmunization

	Frequency of alloimmunization (%)			
Factor	Number of studies	Mean±SD	F/t/r-value	P-value
**Year of publication**				
Before 2010	2	7.97±1.17	1.218^T^	0.331
2010-2015	10	8.52±6.54		
2015-2020	8	14.95±9.96		
After 2020	3	12.70±3.33		
**Sample size (n)**				
<50	6	18.43±10.48	-3.132	0.005*
>50	17	8.72±4.65		
**Region**				
North Africa	5	13.17±11.37	0.542	0.659
West Africa	11	11.80±7.54		
East Africa	6	7.97±2.27		
Southern Africa	1	-		
**Antibody test method**	Number of studies	Mean±SD	F/t/r-value	P-value
CGA	9	11.42±8.30	1.086	0.417
Saline	2	4.15±0.21		
Tube agglutination	4	15.98±16.06		
ICT	2	28.63±24.57		
Gel Card system	2	22.75±8.84		
ID Card micro-typing	1	21.40		
Aut0Vue Bio	1	22.00		
**Age**	23		0.450	0.031*
**Female-male ratio**	20		0.189	0.425

*: statistically significant at α= 0.05

T: epidemiologically significant at difference of ≥4%.

Superscripts: sameness of any superscript means non-epidemiologically significant; difference of superscript means: epidemiologically significant

Antibodies against 35K (10.4%), 59E (17.5%), 45C (13.3%) and 30D (8.9%) antigens accounted for half (50.1%) of the antibody specificities (Table 1).

## Discussion

This systematic review identified and evaluated twenty-three peer-reviewed research papers on RBC alloimmunization in transfused patients with SCD. The pooled prevalence of RBC alloimmunization was 12.1% ((CI 7.7-17.7); 12=91.83; PI = 2.0% to 54.0%)). This is higher than the 6.7% reported by Ngoma et al.^59^ (although this study determined the prevalence of antibodies among transfused non sickle cell disease patients) and the 7.4% reported by Boateng et al.^33^. This result, however, is lower than the alloimmunization rates of some high income countries (22.3% to 58%)^20^,^60^,^61^. This is probably due to a lower antigenic diversity among patients of similar tribal/ethnic origin compared to a higher antigenic diversity between the Caucasian blood donors and recipients who are predominantly of African origin^61^. The lower prevalence in our study can also be explained by the study design, which in most cases is cross-sectional, contrary to studies from well-resourced countries where patients are often routinely tested for antibodies before each transfusion episode. Also, the lower prevalence of alloimmunization obtained in our study compared to high-income countries may also be attributed to under reporting of cases of alloimmunization and limited access to blood for transfusion, which is generally saved for emergency situations since only a few patients can afford regular transfusion. Thus, socioeconomic disparities between African and high-income nations contribute to the differential in red cell alloimmunization among patients with SCD^33^. Given Africa's restricted donor pool, which is heavily influenced by cost and poverty, the guideline^52^,^55^,^62^ that patients with SCD receive transfusions from donors whose RBC antigens have already been matched with those of the patients^63^,^64^ may be impractical. Given the cost implication of red cell alloimmunization, it is needful to re-emphasize Boateng's^33^ recommendation for transfusion safety amongst SCD patients as we expect an increased rate of transfusion amongst SCD, in the coming days. This increased rate of blood transfusion is because of the ageing population of SCD patients due to improved care. Older SCD patients have more complications from multiple organ damage that will require chronic blood transfusion. Routine testing for RBC antibodies and extended blood group phenotyping to identify suitable transfusion units would be an effective strategy to increase transfusion safety for SCD patients in Africa^65^. Comparing our findings to those of two earlier studies, Ngoma et al.^59^ and Boateng et al.^33^, we found an increase in the frequency of red cell alloimmunization among SCD patients in Africa than in multiply transfused non-SCD^28^, ^66^. The plausible explanation for this could be seen from the role of inflammation in the process of alloimmunization. Transfusions during inflammatory episodes such as acute chest syndrome and vasoocclussive crisis in SCD patients were linked to higher rate of alloimmunization^67^. Moreover, in SCD patients, a number of innate immune abnormalities have been found, such as a weakened anti-inflammatory response against extracellular cell free heme. This puts this patient subset at risk of developing a strong immune response against allogeneic determinants on transfused red blood cells, which raises the possibility of further alloimmunization^68^. Furthermore, the increased rate could also be due to a comparatively high lifetime RBC transfusion burden since blood transfusion is a key treatment modality in SCD patients. Nonetheless, alloimmunization rates where donors and SCD patients are more ethnically homogeneous have been reported to be equivalent to alloimmunization frequencies recorded for the general population^25^.

We also observed a marked inconsistency (ups and down) in the distribution of alloimmunization from year to year. The variability in the rate of alloimmunization may be likely due to the variation of test methods used for antibody detection. While some studies use test tube method, others used gel agglutination method which is more sensitive (Table 1). However, in pursuit of the African Union Agenda 2063, an extensive practice of pre-transfusion screening, for which matching of RBC antigens between patients and donors is done, may be achievable in the future and decrease the burden of alloimmunization.

Antibodies to E, D, C, and K antigens accounted for 49.8% of all the antibody specificities, according to our findings. This is consistent with Boateng et al.^33^ as well as da Cunha et al.^69^ both of which are African studies. This suggests that the antibody specificities are relatively consistent. Anti-D antibodies were found in 10.1% of patients in our study, despite the fact that D antigen matching is a common procedure in Africa. People of African origin have a higher prevalence of RHD variants, which may explain the elevated anti-D prevalence.^24^, ^70^ With serology, patients with RHD variants (partial D phenotype) who receive D-positive blood are at risk of producing anti-D^63^. Anti-K antibodies constituted 8.7% of all antibodies. This is lower compared to the prevalence obtained by Boateng et al.^33^.

Consistent with Boateng and colleagues, there were 36 patients with anti-K and of these only three were from West Africa. The anti-K distribution varies substantially across Africa^71^. The heterogeneity in antibody frequency and specificity suggests that more research on blood group antigen variation among several ethnicities is needed. In line with Fasano et al.^63^ and Chou et al.^61^, the rising prevalence of red cell alloimmunization and wide heterogeneity in the antibody specificities necessitates the establishment of antigen typed blood donor database. This may facilitate antibody testing and alloantibody identification^61^, especially if we plan to increase regular blood transfusions in the region in pursuit of the African Union Agenda 2063. As a result, patients with SCD and other patients on chronic blood transfusion would no longer be reliant on expensive, internationally sourced reagent red cells for their RBC antibody testing, as a locally produced alternative could be made available. The study draws its strength from the extensive literature search, independent full-text screening, and extraction. In addition, the studies included in the review are representative of the African region. Nonetheless, several limitations have been identified including high degree of heterogeneity, presence of publication bias, although we conducted meta-regression in attempt to explore the sources thereof. The presence of outlying prevalence values^53^,^57^ constitutes a limitation. Likelihood of developing alloimmunization was dependent on patient's age, number of units of blood received, status of transfused RBCs and antigenic differences between the donor and recipient population^43^. Another limitation was use of small sample size (< 50) with risk of exaggerating the frequency of alloimmunization.

## Conclusion

Within Africa, the frequency of red cell alloimmunization varied considerably and remains low when compared to global prevalence. Alloimmunization in SCD patients in Africa should be given more attention because they are source of morbidity and mortality in this group of patients. In light of the impracticability of RBC antigen matching for all African SCD patients, prior screening and extended crossmatching is essential. To ensure that compatible transfusion units can be quickly selected in an emergency, a database of antigen-typed donors may be vital.
